# Aspiration Pneumonia Incidence and Recurrence in Elderly Stroke Patients: Oral Hygiene and Functional Status

**DOI:** 10.1016/j.identj.2025.103902

**Published:** 2025-09-18

**Authors:** Atsushi Hombo, Koroku Kato, Mai Jokaji-Ishimiya, Yutaka Kobayashi, Takashi Hase, Shuichi Kawashiri

**Affiliations:** a Department of Oral and Maxillofacial Surgery, Kanazawa University Graduate School of Medical Science, Kanazawa, Japan; b Department of Oral and Maxillofacial Surgery, Noto General Hospital, Ishikawa, Japan

**Keywords:** Aspiration pneumonia, Elderly stroke patient, Oral hygiene, Oral function

## Abstract

**Introduction and Aims:**

We investigated the impacts of oral hygiene and oral function on the incidence and recurrence of aspiration pneumonia (AP) in elderly stroke patients.

**Patients and Methods:**

Among the 991elderly patients who underwent eating- and swallowing-function evaluations at the Department of Oral and Maxillofacial Surgery of Noto General Hospital over the years 2013-2017, we retrospectively analyzed the cases of the 559 patients with stroke. We divided them into an aspiration pneumonia (AP) group and a non-pneumonia (NP) group to analyze the relationship between oral hygiene or oral function factors and AP incidence. We further divided the AP patients into those who experienced recurrent pneumonia (rAP group) and those who did not (nrAP group) to examine the association between oral hygiene or oral function factors and AP recurrence.

**Results:**

A univariate analysis comparing the AP and NP groups revealed significant differences in 3 oral hygiene factors: membranous substances, xerostomia, and food residue (*P < .*001, respectively). Regarding oral function factors, only occlusion showed a significant intergroup difference (*P < .*001). A multivariate logistic regression analysis identified age (*P = .*017), tongue fur (*P = .*001), membranous substances (*P = .*005), xerostomia (*P = .*045), food residue (*P < .*001), and occlusion (*P < .*001) as factors influencing the incidence of AP. The univariate analysis comparing the rAP and nrAP groups showed a significant intergroup difference in food residue (*P = .*006) as an oral hygiene factor and significant differences in food bolus formation grade (FBFG) (*P = .*001) and Food Intake LEVEL Scale (FILS) (*P < .*001) as oral function factors. The multivariate logistic regression analysis identified FILS (*P = .*01) as a factor linked to AP recurrence.

**Conclusion:**

Oral hygiene conditions were significantly associated with AP incidence in elderly stroke patients, suggesting that structured oral care may help reduce AP risk. Given the association between oral function and AP recurrence, the importance of functional oral care through rehabilitation was highlighted.

## Introduction

Stroke, a cerebrovascular disease, is a leading cause of death and long-term disability worldwide. Stroke encompasses a range of brain-function disruptions caused by vascular disturbances and is broadly classified into 2 types: ischemic (e.g., cerebral infarction) and hemorrhagic (e.g., intracerebral and subarachnoid hemorrhages). Dysphagia occurs in approximately 40%-70% of stroke patients,^1^ and thus aspiration pneumonia (AP) is 1 of the most common and serious complications in both the acute and recovery phases of stroke.

The primary causative agents of AP are anaerobic bacteria originating from the oral cavity. Poor oral hygiene has been identified as a significant risk factor for AP among elderly individuals, and several studies conducted globally have demonstrated strong associations between oral conditions (e.g., dental caries and periodontal disease) and the incidence of AP.^2^, ^3^, ^4^, ^5^, ^6^, ^7^ Interventional studies have demonstrated that structured oral care can significantly reduce the incidence of AP in older adults, especially in institutional and hospital settings.^2^^,^^3^^,^^5^^,^^8^ Moreover, AP is related not only to swallowing dysfunction but also to oral function overall. Declines in oral function, including decreased saliva production, tongue mobility, and masticatory ability, are known to increase the risk of AP.^9^^,^^10^ Impaired masticatory function due to the tooth loss caused by periodontal disease may also elevate the risk of developing AP.^2^

Despite the growing body of global evidence supporting the link between oral hygiene and respiratory complications in elderly populations, understanding of how oral hygiene and oral function in stroke patients influence the development and recurrence of AP has remained limited, particularly in hospitalized stroke populations where oral care is not routine focus of medical management.

We hypothesized that poor oral hygiene and impaired oral function would be significantly associated with a higher risk of the development and recurrence of AP in elderly stroke patients. We conducted the present study to evaluate the relationships among oral hygiene status, oral and swallowing function, and the incidence and recurrence of AP in a large series of elderly hospitalized stroke patients.

## Patients and methods

### Patients

Among the 991 patients aged ≥65 years who were referred to the Department of Oral and Maxillofacial Surgery at Noto General Hospital in Ishikawa, Japan during the period from 2013 to 2017 for an evaluation of their feeding and swallowing function and who were eligible for examination, we retrospectively analyzed the cases of the 559 elderly patients with a history of stroke in this study. Ethical approval for the study was obtained from the Ethics Committee of the Noto General Hospital (approval no. 2603), and the study protocols were in accordance with relevant guidelines and regulations. Each patient provided written informed consent for the publication of their data and images.

All patient had been evaluated for the presence/absence of AP by internists at Noto General Hospital based on the Nursing and Healthcare-associated Pneumonia (NHCAP) clinical guidelines of the Japanese Respiratory Society.^11^ We divided the 559 patients into 2 groups: those who had been diagnosed with AP (AP group, n = 177) and those who had not (NP group, n=382). We compared the 2 groups with respect to age, sex, oral hygiene factors, and oral function factors.

We further categorized patients of the AP group into 2 subgroups: those who had experienced recurrent pneumonia, defined as at least 1 prior episode of AP before the current assessment (rAP group, n = 89) and those who had not (nrAP group, n = 88). These subgroups were similarly compared in terms of age, sex, underlying disease that led to hospitalization, oral hygiene factors, and oral function factors.

### Assessments

As oral hygiene factors, the following were assessed by a dentist through visual examination using selected indicators from the Revised Oral Assessment Guide (ROAG)^12^: the presence of viscous sputum, membranous substances, xerostomia, tongue fur, mucositis, and food residue. As oral function factors, the following were evaluated: sialorrhea,^13^ occlusion,^13^ the food bolus forming grade (FBFG),^10^ and the Food Intake Level Scale (FILS).^14^ In this context, occlusion was defined as having ≥20 remaining teeth or having functional occlusion with dentures.

**Fig. 1:**
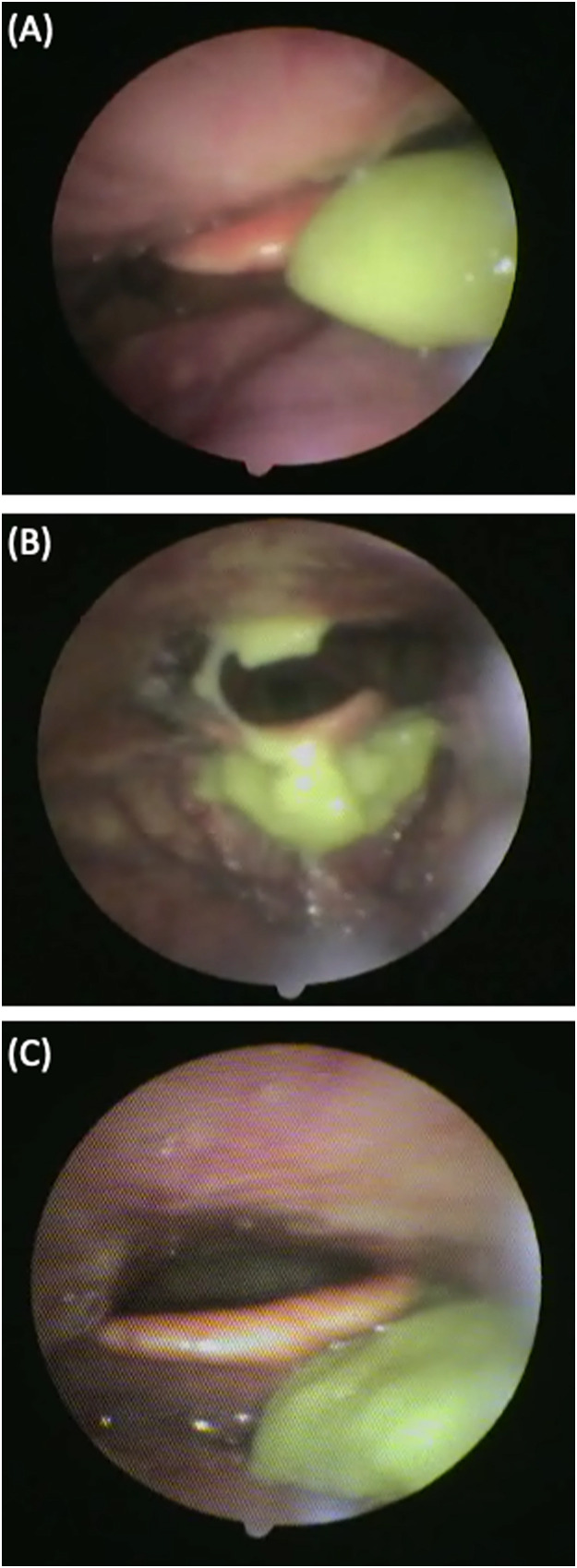
Definitions of the bolus formation grade. (A) Grade 1: Boluses retain their shape. (B) Grade 2: A mixture of bolus sizes. (C) Grade 3: Boluses have been transformed into a homogeneous paste.

The FBFG, introduced by Hase et al. in 2019,^10^ is a system for evaluating bolus formation ability through mastication (Figure 1). It was applied in the present patient series as follows. A nasopharyngeal fiberscope (3.6-mm diameter., ENP TYPE P4; Olympus, Tokyo) was inserted transnasally, and the tip was positioned to allow full observation of the pharyngeal cavity. The patient was instructed to chew and swallow 4 g of 'Process Lead, a chew-and-swallow training food developed by Otsuka Pharmaceutical Factory (Naruto, Japan). A caregiver assisted in administering the food in a single bolus, and the pharyngeal cavity was imaged endoscopically throughout the process.

The shape of the food bolus immediately before swallowing was classified into 3 grades based on the recorded images. Grade 1: The food bolus retained its original shape without any deformation. Grade 2: The food bolus was observed as a mixture of large and small pieces. Grade 3: The food bolus was uniformly deformed into a paste-like consistency. Video endoscopic (VE) images corresponding to each grade of the FBFG are shown in Figure 1. The FBFG classifications were assessed by a team of 3 professionals: a dentist, a registered dietitian, and a certified elderly care worker.

The FILS is a simple tool for assessing food intake ability in individuals with dysphagia. It was proposed in 1993 by the Medical Review Committee of the Japanese Society of Dysphagia Rehabilitation (Table 1)^15^ and is graded as follows. FILS Grades 1-3 (severe type): The individual requires complete nutritional support through alternative feeding. Grades 4-6 (moderate type): The individual requires a combination of oral intake and alternative nutrition. Grades 7-9 (mild type): The individual can maintain sufficient nutrition through oral intake alone. Grade 10 (normal): The individual exhibits normal swallowing function.

**Table 1:** Grade of the food intake level scale (FILS).

Severe (Mainly alternative nutrition)	Grade 1	Oral intake not possible (dysphagia therapy not indicated)
	Grade 2	Only Indirect dysphagia therapy without oral intake allowed
Moderate (Oral intake with alternative nutrition)	Grade 3	Direct dysphagia therapy with oral intake allowed
	Grade 4	Minimal (less than 1 meal) oral intake possible
	Grade 5	Partial (1 or 2 meals a day) oral intake possible
	Grade 6	Oral intake possible for all meals but still require some alternative nutrition
Mild (Oral intake only)	Grade 7	Oral intake of dysphagia diet possible for all meals
	Grade 8	Oral intake of restricted diet possible
		(specific food items that are difficult to swallow avoided)
Normal		
	Grad 9	Oral intake of normal diet possible with supervision
	Grade 10	Normal oral intake and swallowing function

### Statistical analysis

The relationships between the occurrence of AP and factors such as age, sex, underlying disease, oral hygiene factors, and oral function factors were analyzed using the χ²-test, implemented in SPSS version 24 (IBM, Armonk, NY). A multivariate logistic regression analysis was conducted to adjust for potential confounding factors related to age, sex, oral hygiene factors, and oral function factors. In the multivariate analysis, age was dichotomized at 83 years, the median age of the study population. A p-value <.05 was considered significant.

## Results

The total patient series (n = 559) included 298 males and 261 females, with an average age of 80.7 years. The average age of the patients in the NP group (n = 382, –68% of the patient series) was 80.1 years, and the corresponding value in the AP group (n = 177, –32% of the patient series) was significantly higher at 82.4 years (*P = .*002).

Regarding the relationship between oral hygiene factors and AP, significant differences were observed between the AP and NP groups for membranous substances (*P < .*001), xerostomia (*P < .*001), and food residues (*P < .*001). Regarding the relationship between oral function factors and AP, a significant intergroup difference was observed only for occlusion (*P < .*001) (Table 2).

**Table 2:** Univariate analysis of factors associated with the development of aspiration pneumonia (AP) in elderly patients with stroke.

Variables	AP group	NP group	p value
Gender			.907
Male	95	203	
Female	82	179	
Age (Average)	82.4	80.1	.002
Disease			.032
Cerebral infarction	149	289	
Intracerebral hemorrhage	19	75	
Subarachnoid hemorrhage	9	18	
Oral hygene factors			
Viscous sputum			.18
Yes	39	66	
No	138	316	
Membranous substances			<.001
Yes	54	48	
No	123	334	
Xerostomia			<.001
Yes	74	89	
No	103	293	
Tongue fur			.074
Yes	77	136	
No	100	246	
Mucositis			.966
Yes	8	17	
No	169	365	
Food residue			<.001
Yes	111	169	
No	66	213	
Oral function factors			
Sialorrhea			.408
Yes	15	41	
No	162	341	
Occulusion			<.001
Yes	67	208	
No	110	174	
FBFG			.844
I	38	74	
Ⅱ	62	136	
III	77	172	
FILS			.883
Sever	58	120	
Moderate	117	256	
Mild	2	6	

A multivariate logistic regression analysis was conducted to adjust for significant factors related to the incidence of AP, and the odds ratios (ORs) and 95% confidence intervals (CIs) were calculated. As shown in Table 3, the analysis identified the following as risk factors for AP: age ≥83 years (OR 1.65; 95%CI, 1.06-2.57; *P = .*028), presence of membranous substances (OR 2.62; 95%CI, 1.33-5.2; *P = .*006), presence of food residues (OR 3.5; 95%CI, 2.25-5.44; *P < .*001), and absence of occlusion (OR 1.96; 95%CI, 1.3-2.93; *P = .*001).

**Table 3:** Logistic regression analysis of factors associated with the development of aspiration pneumonia (AP) in the study population.

Variables	Groups	95% Confidence interval	p value	Odds ratio
Age	<83 / 83≤	1.06-2.57	.028	1.65
Membranous Substances	+ / –	1.33-5.2	.006	2.62
Xerostomia	+ / –	0.825-2.77	.181	1.51
Food residue	+ / –	2.25-5.44	<.001	3.5
Occulusion	+ / –	1.3-2.93	.001	1.96

*The cut-off value of 83 years was determined based on the median age of the study population.

We next divided the 177 patients in the AP group into an nrAP group (n=88) who had experienced AP only once, without recurrence, and a rAP group (n=89) who had experienced recurrent AP. The average age of the nrAP group was 81.1 years, and that of the rAP group was significantly higher at 83.4 years (*P = .*029). We analyzed the relationships between recurrent AP and the patients’ age, sex, oral hygiene factors, and oral function factors to identify risk factors.

As sown in Table 4, analysis of the relationship between oral hygiene factors and AP recurrence revealed a significant difference in present of food residues between the nrAP and rAP groups (*P = .*006). Regarding the relationship between oral function factors and AP recurrence, significant intergroup differences were observed for FGFG (*P < .*001) and FILS (*P < .*001). Finally, the multivariate logistic regression analysis identified a single risk factor for recurrent AP: severe FILS (OR 2.15, 95%CI, 1.19-3.88; *P = .*011) (Table 5).

**Table 4:** Univariate analysis of factors associated with recurrence of aspiration pneumonia (AP) in patients with recurrence (rAP group) and without reccurrence (nrAP group).

Variables	rAP group	nrAP group	p value
Gender			.245
Male	49	56	
Female	40	32	
Age (Average)	83.4	81.1	.029
Disease			.575
Cerebral infarction	76	73	
Intracerebral hemorrhage	10	9	
Subarachnoid hemorrhage	3	6	
Oral hygene factors			
Viscous sputum			.344
Yes	17	22	
No	72	66	
Membranous Substances			.353
Yes	30	24	
No	59	64	
Xerostomia			.144
Yes	42	32	
No	47	56	
Tongue fur			.932
Yes	39	38	
No	50	50	
Mucositis			.344
Yes	17	22	
No	72	66	
Food residue			.006
Yes	47	64	
No	42	24	
Oral function factors			
Sialorrhea			.845
Yes	8	7	
No	81	81	
Occulusion			.923
Yes	34	33	
No	55	55	
FBFG			<.001
I	30	8	
Ⅱ	30	32	
III	29	48	
FILS			<.001
Sever	41	17	
Moderate	45	71	
Mild	3	0

**Table 5:** Logistic regression analysis for factors associated with the recurrence of aspiration pneumonia (AP) in the study population.

Variables	Groups	95% Confidence interval	p value	Odds ratio
				
Age	<83 / 83≤	0.675-2.57	.418	1.32
Food residue	+ / –	0.258-1.05	.068	0.521
FBFG	I,II / III	0.547-2.63	.652	1.2
FILS	Mild, Mod / Sev	1.31-7.14	.01	3.06

*The cut-off value of 83 years was determined based on the median age of the study population.

## Discussion

Aspiration of food or saliva can lead to the development of AP. The proportion of patients diagnosed with AP in Japan is higher than those in other countries, making it a significant health concern in Japan, particularly among the elderly population.^2^^,^^16^ The incidence of AP has been shown to increase with age among patients hospitalized for pneumonia, with prevalence rates at 30% in patients aged 50-59 years, 50% in those aged 60-69 years, and 80% in patients aged ≥70 years.^1^ According to the statistics compiled in 2023 by Japan's Ministry of Internal Affairs and Communications, the aging rate in Japan (i.e., the proportion of people aged ≥65) reached a record high of 29.1% that year and was the highest in the world. As the aging rate in Japan is projected to continue increasing, AP is expected to remain a critical public health issue not only at the individual level but also for society as a whole.^2^

Aspiration pneumonia is a form of pneumonia caused by inhaling bacteria-laden material—such as saliva, oropharyngeal secretions, or gastric contents—into the lungs.^15^^,^^17^^,^^18^ Oral hygiene has been identified as a contributing factor in the development of AP, since poor oral hygene leads to the proliferation of various bacterial species in the oral cavity, which can be aspirated with saliva and lead to infection.^17^^,^^19^, ^20^, ^21^

Saliva plays a crucial role in inhibiting bacterial overgrowth by exerting antibacterial effects that help maintain the oral microbiome and providing a self-cleansing action that washes away food debris.^22^ However, in elderly individuals with multiple comorbidities, salivary secretion can decline due to the side effects of medications and age-related changes.^14^^,^^23^, ^24^, ^25^ This reduction compromises both the antibacterial and self-cleansing functions of saliva, creating an environment that is conducive to bacterial proliferation in the oral cavity. Under such poor oral hygiene conditions, the aspiration of oral bacteria may occur not only during meals but also through silent aspiration during sleep, leading to AP.^26^

Suzuki et al. reported that in elderly patients hospitalized with AP, oral functions and swallowing ability—as indicated by denture use and scores on a validated oral intake scale—affect patient survival rates.^27^ Moreover, AP can occur not only in elderly individuals with impaired swallowing function but also in patients with neurological conditions, such as Parkinson’s disease or the sequelae of stroke, as the result of a reduced cough reflex and microbial retention in the lower respiratory tract.^16^ It has thus been suggested that not only oral hygiene but also oral and swallowing functions influence the incidence of AP.^14^

Although early screening for dysphagia and oral care in patients who have had an acute stroke significantly reduces the incidence of AP,^28^ our search of the relevant literature identified no investigation of the relationship between the incidence or recurrence of AP and oral hygiene or oral function. To our knowledge, this is the first published investigation of the impacts of oral hygiene and oral function on the incidence and recurrence of AP in elderly stroke patients.

Our comparison of the AP and NP groups revealed significant intergroup differences in 3 oral hygiene factors—membranous substances, xerostomia, and food residue—and in 1 oral function factor: occlusion. The multivariate analysis identified membranous substances, food residue, and occlusion as risk factors for the development of AP. These results suggest that reduced salivary self-cleansing due to xerostomia leads to the accumulation of membranous substances as well as the retention of food debris, which in turn promotes bacterial overgrowth, thereby contributing to the incidence of AP. Moreover, inadequate mastication impairs proper bolus formation, which can result in the aspiration of poorly formed food boluses and, ultimately, the development of pneumonia.

Poor oral hygiene has been reported to be a direct risk factor for AP in elderly individuals,^2^^,^^5^ and the results of our present examination of elderly stroke patients support this concept. A prospective cohort study of occlusion and AP indicated that the use of dentures may reduce the risk of AP development.^17^ Considering our present findings, oral hygiene and occlusal status appear to play important roles in the development of AP. These results suggest that improving oral hygiene and maintaining proper masticatory function may contribute to the prevention of AP in elderly individuals who have experienced a stroke.

The results of our comparison of the rAP and nrAP groups demonstrated that, among the oral hygiene factors, only the presence of food residues showed a significant intergroup difference. Among the oral function factors investigated in this study, the FBFG and FILS results were found to be significantly associated with AP recurrence. Finally, the multivariate analysis identified FBFG and FILS grades as risk factors for AP recurrence. These findings suggest that oral function plays a more significant role than oral hygiene in the recurrence of AP.

Yamano et al. reached a similar conclusion, reporting that the assessment of swallowing function was more critical than the assessment of oral hygiene for predicting the risk of recurrent AP in their elderly patient cohort,^29^ Hase et al. demonstrated that inadequate bolus formation can result in food retention in the hypopharynx, leading to aspiration during feeding or silent aspiration, thereby increasing the likelihood of pneumonia.^10^ In summary, in order to prevent and reduce the recurrence of AP, maintaining oral hygiene is essential, and improving oral function is even more important. Elderly patients who have suffered from AP often experience a temporary inability to consume food orally, which can lead to a decline in oral function. An early resumption of oral intake thus requires screening tests for bolus formation and swallowing function, followed by appropriate rehabilitative interventions based on the screening results.

Recent international studies have emphasized the importance of implementing structured oral hygiene care programs in stroke rehabilitation settings. For example, the Royal College of Physicians in the UK and the American Heart Association/American Stroke Association recommend early dysphagia screening and integrated oral care as essential components of stroke management protocols.^30^^,^^31^ Randomized controlled trials conducted in Europe and North America demonstrated that multidisciplinary oral care interventions involving nurses, dental hygienists, and speech therapists can significantly reduce the incidence of AP and improve overall outcomes in post-stroke patients.^32^^,^^33^

Our present study adds to this international body of evidence by focusing specifically on the impact of oral hygiene and oral function on the incidence and recurrence of AP among elderly stroke patients in Japan. Our finding that oral hygiene is more closely associated with AP onset whereas oral function plays a greater role in recurrence offers a nuanced understanding that may help tailor phase-specific interventions: e.g., hygiene-focused care in acute settings and functional rehabilitation during recovery.

These results underscore the need to integrate global best practices such as standardized oral assessment and team-based oral care protocols into domestic stroke-care models. Adapting such programs to Japan’s aging population and healthcare infrastructure may further improve outcomes for stroke survivors who are at risk of AP.

## Conclusion

The results of this study suggest that oral hygiene conditions are strongly associated with the onset of AP in elderly stroke patients. The recurrence of AP in such patients appears to be more strongly associated with oral function, particularly bolus formation ability, than with oral hygiene. These findings highlight the importance of considering both oral hygiene and oral function when assessing the risk of AP development and recurrence. Further interventional studies are needed to investigate the potential benefits of targeted oral care strategies in elderly stroke patients.

## Availability of data and materials

The datasets used and/or analyzed in this study are available from the corresponding author upon reasonable request.

## Ethics statement

The study was approved by the Ethics Committee of Noto General Hospital (approval no.2603).

## Consent for publication

All authors consent to the publication of this manuscript.

## Consent to participate

All patients provided fully informed written consent for the publication of their data and images.

## Funding

This research did not receive any specific grant from funding agencies in the public, commercial, or not-for-profit sectors.

## Author contributions statement

AH, TH and MI were involved in the conception, design, analysis and/or interpretation of the data. SK and YK drafted the manuscript and revised it critically for intellectual content. KK made the final approval of the version to be published. All authors agree to be accountable for all aspects of the work.

## Conflict of interest

None disclosed.
